# Thyroid Abscess: Challenges in Diagnosis and Management

**DOI:** 10.1177/2324709618778709

**Published:** 2018-05-25

**Authors:** Niharika Yedla, Daniela Pirela, Alex Manzano, Claudio Tuda, Saberio Lo Presti

**Affiliations:** 1 Mount Sinai Medical Center, Miami Beach, FL, USA; 2 University of Miami, Miami, FL, USA

**Keywords:** acute suppurative thyroiditis, thyroid abscess, methicillin-resistant *Staphylococcus aureus*

## Abstract

Thyroid abscess is an uncommon infectious pathology. The thyroid is highly resistant to infection due to high iodine content, capsular encasement, and rich vascularity. Acute suppurative thyroiditis represents <1% of thyroid diseases that could potentially become a life-threatening endocrine emergency. A 48-year-old woman with AIDS presented with 3 days of fever, tender neck swelling, and methicillin-resistant *Staphylococcus aureus* bacteremia. Apart from leukocytosis, initial laboratory values including thyroid function tests were normal. The initial plain computed tomography scan of the neck and ultrasound scan of the neck were inconclusive as well. By day 4, she worsened, and on repeat computed tomography scan of the neck with contrast, multiloculated abscesses in the thyroid and retro pharynx were seen, which needed emergent drainage. Acute suppurative thyroiditis, a rare disease, occurs in patients with either preexisting disorders of the thyroid or in the immunocompromised. The most common pathogen is *Staphylococcus aureus*. In our case, we highlight the fact that initial imaging may be negative in the early stages of acute suppurative thyroiditis and lead to an erroneous diagnosis of subacute thyroiditis. There are less than 5 cases of methicillin-resistant *Staphylococcus aureus* suppurative thyroiditis reported.

## Introduction

The thyroid gland is essentially protected from being seeded by infections due to its anatomy. It is surrounded by a capsule, and it has excellent blood supply and lymphatic drainage. The iodine content of the gland also prevents infections. Even among those with impaired immune systems, acute suppurative thyroiditis (AST) is a rare disease with incidence being less than 1% of all thyroid diseases.^1^ It is most commonly caused by gram-positive aerobes, *Staphylococcus aureus* being the most prevalent, but there are also reports of gram-negative organisms and fungi.^1^ Extensive PubMed search brought to light less than 5 reported cases of methicillin-resistant *Staphylococcus aureus* (MRSA) thyroid abscesses.^2-5^ AST is a life-threatening endocrine emergency. It presents with fever, neck pain, and swelling. Thyroid hormones may be normal or elevated, but they do not aid in diagnosis or management.^6^ Ultrasound of the thyroid is the suggested imaging modality of choice. Computed tomography (CT) scan and magnetic resonance imaging are generally not needed.^7^ Due to its rarity, only case reports and case series are available to guide management. Traditional treatment includes surgical drainage and systemic antibiotics.

## Case Presentation

We present a 48-year-old woman with significant past medical history of uncontrolled diabetes type 2, former intravenous drug user, human immunodeficiency virus, noncompliant with antiretroviral therapy, untreated hepatitis C virus, and epilepsy who presented with a severe sore throat. She reported these symptoms for the last 3 days, which were gradual in onset, mild in intensity, accompanied by nausea and difficulty swallowing, and that progressed within hours into difficulty breathing. She denied any fevers or chills, cough, runny nose or earaches, any palpitations, anxiety, heat intolerance, or weight loss.

On physical examination, she was noted to be afebrile, tachycardia into 120s, dyspneic, and hypotensive. She had a tender anterior neck swelling without palpable lymph nodes. No exudates were noted in the oropharynx. She had bilateral wheezing without stridor. The rest of the systemic examination was unremarkable.

Laboratory tests revealed leukocytosis with bands; platelets were reduced to 30 000/mL. Chemistry revealed sodium of 135 mmol/L, potassium of 2.9 mmol/L, blood urea nitrogen of 32 mg/dL, and creatinine of 2.67 mg/dL. The thyroid function test revealed thyroid-stimulating hormone to be within the normal range at 3.740 µIU/mL with elevated free thyroxine (T4) and free tri-iodothyronine (T3) at 2.33 µg/dL and 5.16 ng/dL, respectively. Antithyroperoxidase antibodies and thyroglobulin antibodies were negative. Her CD4 count was 7 cells/mm^3^, with 873 000 viral copies/mL.

Urine analysis revealed significant pyuria and bacteriuria. Due to the acute kidney injury, a noncontrast CT scan of the neck was done, which showed fat stranding in the anterior cervical region and around the thyroid gland with marked narrowing of the subglottic airway. An ultrasound of the thyroid gland failed to reveal any obvious fluid collections to suggest an abscess or features of AST-like increased vascularity. Based on these findings, we considered subacute thyroiditis as the probable differential.

She was admitted to the intensive care unit and placed on broad-spectrum antibiotics, piperacillin-tazobactam with vancomycin. Highly active antiretroviral therapy along with bactrim and azithromycin prophylaxis, acyclovir, and fluconazole for opportunistic infections were started.

**Figure 1 and 2.**
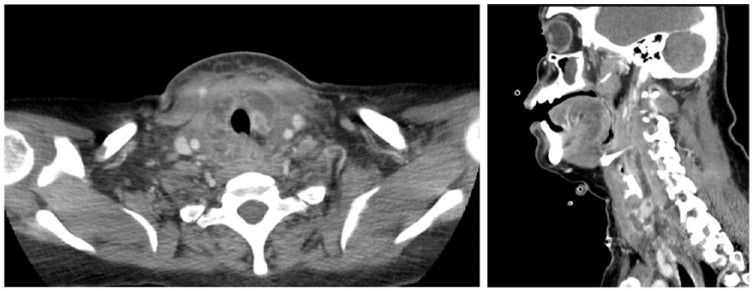
Computed tomography of the neck with contrast with extensive multilocular ring-enhancing lesions around the thyroid cartilage, extending superiorly into the retropharyngeal and prevertebral space at the C7-T1 level and extending posteriorly to the para-pharyngeal region.

Her blood cultures came back positive with MRSA; hence, only vancomycin was continued. A transthoracic echocardiography and carotid Doppler ultrasound were inconclusive. Urine cultures also grew MRSA. By day 4 of hospitalization, the patient had worsening neck pain and stridor. She was taken for a stat contrast CT-neck, which revealed multiple abscesses in the thyroid as well as in the retropharyngeal space (Figures 1 and 2). She was taken to the operating room for transoral and transcervical drainage of the thyroid abscesses. She was intubated postprocedure for possible airway edema and observed in the intensive care unit. The cultures from the abscesses also grew MRSA. In the setting of persistent bacteremia and suspicion for endocarditis, a transesophageal echocardiography was done with no evidence of vegetations. She was successfully extubated and her repeat thyroid hormone studies postdrainage were in euthyroid ranges. However, by day 7, she again developed worsening neck pain, fevers, and lactic acidosis. Repeat CT scan of the neck showed re-accumulation of abscesses. She was taken to the operating room again for surgical exploration and drainage. She improved postprocedure. She was given a total 14 days intravenous vancomycin. She was successfully discharged from the hospital.

## Conclusion

In conclusion, we present a very rare presentation of MRSA suppurative thyroiditis with bacteremia in an immunosuppressed individual with AIDS. Initial imaging studies may not always be conclusive in the early phase of AST prior to abscess formation. Hence, we emphasize the need to pursue the diagnosis of AST when clinically prompted. Initial imaging studies may not always be conclusive.

## Discussion

Acute infection of the thyroid is a less seen diagnosis, mostly so as the gland is inherently protected. The encapsulation, rich blood and lymphatic supply, and iodine content inside offer the gland protection from being seeded. However, it is a differential to be kept in mind in a patient with a tender neck.^8^

In children, the presence of a pyriform sinus may predispose to this infection, whereas in adults a compromised immune state, especially acquired immunodeficiency syndrome (AIDS), can do the same.^1^ History of previous thyroid disease is another risk factor.^8,9^

Major pathogens are *Staphylococcus* and *Streptococcus* species about 35% to 40% of the time. Gram-negative organisms cause about 25% cases, whereas anaerobes around 9% to 12%. The rest being fungal etiologies.^1,5^ Mycobacterial and fungal cases tend to be more common in immunocompromised patients and are chronic in nature, while bacterial causes are more acute.^10,11^

Yu et al^6^ performed a review of 191 cases from 1980 to April 1997 and compared it with a review of 224 cases (1900-1980). They found that as the numbers of immunocompromised patients has been increasing, cases of suppurative thyroiditis are increasing. Interestingly, they found bacterial thyroiditis to present in a euthyroid state (83.1%), while mycobacterial were more hyperthyroid (50%) and fungal more hypothyroid (62.5%).^6^ In our case immunocompromised state seems to be the risk factor.

Early diagnosis is important as the disease can progress rapidly and may prove fatal. Ultrasound is the preferred imaging technique for diagnosis of thyroid diseases.^12^ It also offers the advantage of needle aspiration. Unless inconclusive, one need not obtain a CT scan or magnetic resonance imaging.^7^ If an iodine scan is done, abscess areas may appear as cold.^9^ Antimicrobials and surgical drainage of the abscess is the treatment of choice. In cases with pyriform sinus fistulas, chemocauterization techniques are being utilized, due to being less invasive.^13^

As per our literature search, less than 5 previous cases of MRSA thyroiditis have been reported.^2-5^ Lethert et al^2^ reported a case, with unknown source, diagnosed with ultrasound and needle aspiration found to have thyrotoxicosis who was successfully treated with vancomycin. Elorza et al^3^ described a case where a man presented 3 months after total cystectomy for invasive bladder cancer with anterior neck pain. He was diagnosed with a CT scan and treated with surgical drainage. Cabizuca et al^5^ reported a case of thyroid abscess seeded from infective endocarditis.

In our case, MRSA grew in wound cultures as well as blood and urine cultures. The proposed causal web here is based on immunocompromised state and history of intravenous drug use. We suspect skin colonization with MRSA, which entered her blood stream and caused bacteremia. The same bacteria spilled into the urine and seeded the thyroid with the immunosuppressed state favoring the infection.

Due to the initial negative imaging studies and thyroid abscesses being a rare entity, reaching a diagnosis was a challenge. Since this is a life-threatening disease, early recognition and high clinical suspicion is essential. Another rare phenomenon to highlight from our case was the re-accumulation of the abscess after initial drainage. We feel that it is important to recognize AST and associated early abscess formation in the differential diagnosis of anterior neck pain in immunocompromised patients.

## References

[1] Paes JE Burman KD Cohen J et al Acute bacterial suppurative thyroiditis: a clinical review and expert opinion. Thyroid. 2010;20:247-255. 20144025 10.1089/thy.2008.0146

[2] Lethert K Bowerman J Pont A Earle A Garcia-Kennedy R Methicillin-resistant *Staphylococcus aureus* suppurative thyroiditis with thyrotoxicosis. Am J Med. 2006;119:e1-e2. 10.1016/j.amjmed.2006.03.016 17071148

[3] Elorza JL Echenique-Elizonda M Acute suppurative thyroiditis. J Am Coll Surg. 2002;195:729-730. 12437264 10.1016/s1072-7515(02)01322-4

[4] Brook I Role of methicillin-resistant *Staphylococcus aureus* in head and neck infections. J Laryngol Otol. 2009;123:1301-1307. 19664316 10.1017/S0022215109990624

[5] Cabizuca CA Bulzico DA de Almeida MH Conceição FL Vaisman M Acute thyroiditis due to septic emboli derived from infective endocarditis. Postgrad Med J. 2008;84:445-446. 18832408 10.1136/pgmj.2008.067850

[6] Yu EH Ko WC Chuang YC Wu TJ Suppurative *Acinetobacter baumanii* thyroiditis with bacteremic pneumonia: case report and review. Clin Infect Dis. 1998;27:1286-1290. 9827283 10.1086/514998

[7] Naik KS Bury RF Imaging the thyroid. Clin Radiol. 1998;53:630-639. 9766716 10.1016/s0009-9260(98)80289-4

[8] Mohi GK Datta P Chander J Das A *Citrobacter freundii* as a cause of acute suppurative thyroiditis in an immunocompetent adult female. Indian J Pathol Microbiol. 2017;60:282-284. 28631657 10.4103/0377-4929.208400

[9] Pearce EN Farwell AP Braverman LE Thyroiditis. N Engl J Med. 2003;348:2646-2655. 12826640 10.1056/NEJMra021194

[10] McAninch EA Xu C Lagari VS Kim BW Coccidiomycosis thyroiditis in an immunocompromised host post-transplant: case report and literature review. J Clin Endocrinol Metab. 2014;99:1537-1542. 24606101 10.1210/jc.2013-4373

[11] Chaudhary V Bano S Thyroid ultrasound. Indian J Endocrinol Metab. 2013;17:219-227. doi:10.4103/2230-8210.109667. 23776892 PMC3683194

[12] Das DK Pant CS Chachra KL Gupta AK Fine needle aspiration cytology diagnosis of tuberculous thyroiditis. A report of eight cases. Acta Cytol. 1992;36:517-522. 1636345

[13] Miyauchi A Inoue H Tomoda C Amino N Evaluation of chemocauterization treatment for obliteration of pyriform sinus fistula as a route of infection causing acute suppurative thyroiditis. Thyroid. 2009;19:789-793. 19508119 10.1089/thy.2009.0015

